# Are we researching the right questions? Bibliometric analysis of undergraduate nursing thesis alignment with Peru's health priorities

**DOI:** 10.3389/frma.2026.1738032

**Published:** 2026-02-12

**Authors:** Carla Cuya-Zevallos, Dely Lazo-Barreda, Mirta Cardeña-Valverde, Teresa Chocano-Rosas, Teddy Salazar, Nika Corvacho, Camila Monroy, Ana Pinto, Alvaro Veleto

**Affiliations:** 1 Faculty of Nursing, Universidad Católica de Santa María, Arequipa, Peru; 2 School of Systems Engineering, Universidad Católica de Santa María, Arequipa, Peru; 3 Faculty of Human Medicine, Universidad Católica de Santa María, Arequipa, Peru

**Keywords:** bibliometrics, health priorities, higher education, nursing, research design, social impact, Sustainable Development, thesis

## Abstract

**Background:**

In resource-constrained settings, university research is increasingly expected to demonstrate alignment with national health agendas. In Peru, undergraduate thesis completion is mandatory for professional nursing licensure, generating substantial research output; however, the thematic orientation of this production has not been systematically examined.

**Objective:**

To evaluate the thematic alignment of Peruvian undergraduate nursing theses with Sustainable Development Goals and National Health Research Priorities through comprehensive bibliometric analysis.

**Methods:**

A bibliometric analysis was conducted on 6,157 undergraduate nursing theses produced between 2020 and 2025 across 38 SUNEDU-licensed universities, retrieved from RENATI, ALICIA, and institutional repositories. Descriptive, temporal, and thematic analyses were performed using the Bibliometrix package (R). Keyword co-occurrence networks were generated using VOSviewer. Thematic Alignment Indices (TAI) were calculated to quantify alignment with the 17 SDGs and 53 National Health Research Priorities.

**Results:**

Thematic distribution showed a strong concentration in SDG 3 (Good Health and Well-being, 83.06%), while alignment with National Health Research Priorities was more evenly distributed, with the five most frequent priorities accounting for 49.63% of theses. Research predominantly addressed health habits and lifestyles (14.65%), maternal–child malnutrition and anemia (11.66%), mental health (8.45%), and cardiovascular and metabolic diseases (8.38%). Quantitative methodologies predominated, with limited intervention research. Keyword analysis identified four thematic clusters with minimal integration across domains.

**Conclusions:**

This study provides a descriptive overview of undergraduate nursing research trends in Peru, highlighting thematic concentration in health- related domains and variability in methodological reporting. The findings reflect nursing's disciplinary focus and curricular context, while offering a replicable bibliometric approach for examining undergraduate research orientation in settings with mandatory thesis requirements and limited research investment.

## 1 Introduction

University research has traditionally been regarded as a fundamental pillar for the generation of knowledge, operating under a positivist paradigm in which excellence was measured primarily by scientific publications and academic citations (Hama and Elnadeef, 2025; Gibbons et al., 1994; Verma and Verma, 2023). In this model, it was assumed that social benefits would arise almost automatically from the production of high-quality science, without requiring explicit demonstration of its concrete impact on society (Belcher et al., 2020).

This approach has been progressively supplanted by growing global expectations requiring higher education institutions to play a more active role in addressing contemporary social challenges (Fecher and Hebing, 2021), such as climate change, global health crises, and the achievement of the Sustainable Development Goals (SDGs) (United Nations Organization, n.d.). This transformation requires research to demonstrate relevance and yield measurable benefits beyond academia, particularly in resource-constrained settings where research investment remains limited (Belcher et al., 2020; Bornmann, 2012; Smolentseva, 2022).

Latin America exemplifies this challenge, with significant gaps in science and technology investment (Ciocca and Delgado, 2017; OECD, 2021). Peru invests merely 0.15% of gross domestic product in research and development, maintaining a density of only 0.2 researchers per thousand economically active individuals (Cervantes Liñán et al., 2019; Consejo Nacional de Ciencia Tecnología e Innovación, 2024). This severely limits capacity to generate knowledge addressing specific national needs, particularly in health, where critical epidemiological challenges persist (Rios-Blancas et al., 2023). National health indicators reveal an epidemiological transition with profound implications: non-communicable diseases account for 58.5% of annual disease burden, with projections indicating 1.7 million Peruvians affected by diabetes and over 5.5 million with hypertension by 2025. Simultaneously, acute respiratory infections in children, mental health disorders, and infectious diseases including tuberculosis and dengue continue threatening public health (Rios-Blancas et al., 2023; Ministerio de Salud, 2019).

Within this constrained ecosystem, universities concentrate most scientific output, and undergraduate theses represent substantial but potentially underutilized knowledge sources (Pereyra-Elías et al., 2014; Henein and Ells, 2021). Peruvian University Law 30220 (Congreso de la República-Perú, 2022) mandates thesis completion for professional licensure, generating considerable research volume, yet limited integration between academic products and national health agendas (MINSA, 2024) represents both lost intellectual resources and restricted potential impact on public health. Nursing emerges as particularly relevant given its comprehensive approach and community proximity, uniquely positioned to conduct research responding to population needs across health promotion, disease prevention, diagnosis, treatment, and rehabilitation (Pelayo González-Torre, 2019; Vergara-Mejía et al., 2022; Haakenstad et al., 2022).

However, scientific output in health demonstrates considerable gaps regarding volume, quality, and geographic representation, with high-income country institutions dominating while Global South contributions—crucial for understanding diverse epidemiological and cultural realities—remain scarce (Ciocca and Delgado, 2017; Daza-Orozco et al., 2024; Achury-Saldaña et al., 2022). Previous studies confirm that Latin American nursing research concentrates on descriptive approaches, with limited progression to intervention research and weak linkages to national research priorities (Wang et al., 2022; Jaíne Rorato et al., 2021; Riegel et al., 2021; Moromi et al., 2022). In Peru specifically, only 17.2% of undergraduate nursing theses (2006–2020) translated into indexed publications, reflecting methodological training deficiencies and institutional support limitations (Ñique et al., 2021).

The study aimed to quantify the volume and characterize the methodological features of this research corpus; systematically measure its thematic alignment with two strategic frameworks: the United Nations Sustainable Development Goals (SDGs) and Peru's National Health Research Priorities; and identify research patterns, strategic gaps, and opportunities to better integrate formative nursing research with public health policy and national and global development agendas.

## 2 Material and methods

### 2.1 Study design

This study employed descriptive bibliometric analysis to systematically evaluate undergraduate nursing theses registered from licensed Peruvian universities (SUNEDU, 2025) between January 1, 2020 to July 31, 2025. Bibliometric analysis quantitatively examines scholarly publications to identify patterns, trends, and thematic structures in research production. The descriptive-analytical approach characterizes research corpus composition, temporal patterns, methodological approaches, and thematic distributions.

### 2.2 Search strategy, data sources, and corpus construction

#### 2.2.1 Search strategy and data sources

The study corpus was constructed through a systematic, multi-stage process of manual data extraction and verification from three primary Peruvian sources. A significant technical challenge was the absence of Digital Object Identifiers (DOIs) for Peruvian undergraduate theses. This limitation necessitated the development of robust, alternative methods for deduplication and record matching, as detailed in the following procedure.

Data extraction was conducted as follows:

1. RENATI (National Registry of Research Works) (SUNEDU, n.d.): We accessed Peru's centralized thesis registry maintained by SUNEDU. Using its filters, we selected: University; Academic Area = “Nursing”; Academic Level = “Undergraduate Thesis”; and Publication Year = “2020 to 2025”. This search, executed on July 31, 2025, yielded 4,102 records.
2. ALICIA (Access to Scientific Peruvian Information Portal) (Concytec, n.d.): Manual searches were performed using the portal's advanced search interface with the filters: Document Type = “Undergraduate Thesis”; Discipline = “Nursing”; and Publication Year = “2020–2025”. This process, completed on July 31, 2025, retrieved 6,043 records.
3. Institutional Repositories: We manually accessed the digital repositories of all 38 SUNEDU-licensed universities. For each, we navigated to the nursing faculty section and applied filters for undergraduate theses, document type = Thesis, degree = undergraduate, year = 2020 and 2025. This process, completed on July 31, 2025, retrieved 6,020 records.

When a thesis could not be accessed directly through RENATI and ALICIA (due to broken links or access restrictions), a cross-referencing protocol with institutional repository was performed. This triangulation approach maximized corpus completeness while ensuring all included theses had verifiable full-text accessibility.

#### 2.2.2 Identification and deduplication of records

**Figure 1:**
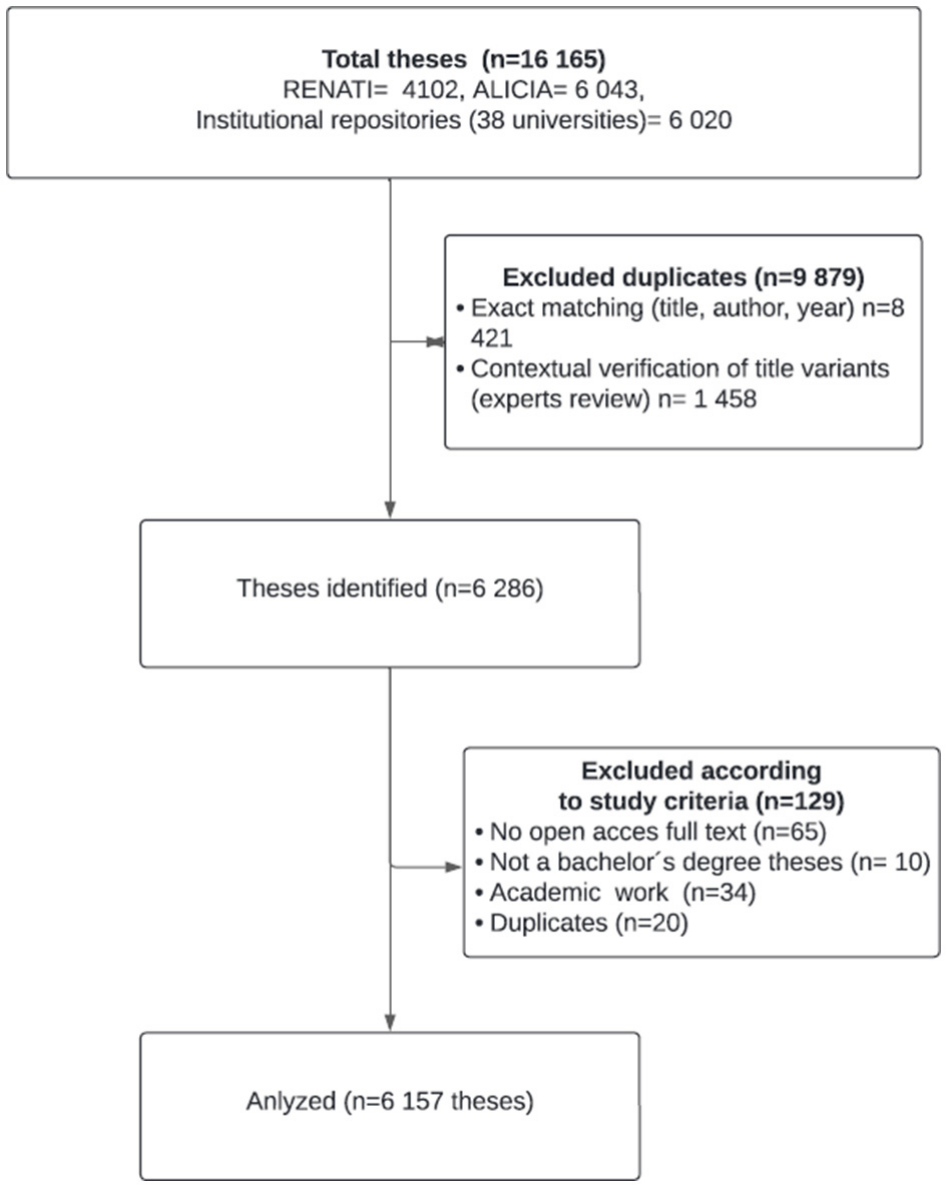
Flow diagram summarizing the selection process.

The initial searches across RENATI, ALICIA, and 38 institutional repositories yielded 16 165 raw records. To ensure a unique and accurate corpus, a rigorous manual deduplication and verification protocol was implemented (Figure 1) on the aggregated records:

1. **Manual Exact Matching:** Records were consolidated into a master spreadsheet and systematically sorted by key fields including title, author, university, publication year, keywords, and abstract. Using manual verification and cell filtering in Microsoft Excel, researchers identified entries with completely identical metadata across repositories. This initial process confirmed 8 421 duplicate entries for consolidation.
2. **Contextual Verification of Titles with Variants:** For records where titles exhibited minor orthographic variations, punctuation differences, or subtle wording changes, a refined manual procedure was employed. Researchers used Excel's filtering tools to isolate clusters of similar titles. Subsequently, three subject-matter experts performed a side-by-side comparison, critically contrasting the potentially matching titles with the associated author names and institutional affiliations to determine if they constituted duplicate records of the same work. This expert contextual assessment identified 1 458 additional duplicates.
3. **Quality Control and Final Inclusion:** Following deduplication, a final pool of 6 286 unique theses was obtained. A subsequent manual verification excluded 129 theses:

- Theses without open access (65)
- Not bachelor's degree theses (10)
- Academic works (34)
- Duplicates (20)

This final curation resulted in analytic corpus of 6 157 unique undergraduate nursing theses.

The selection was restricted to works available in full text and classified explicitly under the nursing academic area, and theses registered from January 2020 to July 2025. This research focused on undergraduate nursing theses submitted to obtain licentiate degrees as stipulated by Peruvian University Law 30220 (Congreso de la República-Perú, 2022), which mandates that students submit a research thesis as a graduation requirement. This licentiate qualification permits graduates to practice professionally within various institutions governed by the Peruvian health system under the General Health Law (MINSA, n.d.).

### 2.3 Data analysis and visualization

All analyses were conducted using R (version 4.3.1) within RStudio (version 2023.09.1) (K-Synth Srl team, 2025). Bibliometric analyses and descriptive statistics were performed using the Bibliometrix package, while data handling and visualization were supported by dplyr and ggplot2. The analytical strategy combined performance analysis and science mapping to characterize the volume, thematic orientation, methodological profile, and conceptual structure of undergraduate nursing theses.

### 2.4 Thematic alignment classification procedure

Thematic alignment with the 17 Sustainable Development Goals (SDGs) and Peru's 53 National Health Research Priorities was determined through a structured, real-time consensus process. A panel of four experts, all formally trained in SDG frameworks and possessing expertise in the Peruvian health system, conducted this work during synchronous virtual sessions over 1 month.

The classification was based on the primary research objective stated in each thesis. For most records, the objective was sourced from the title and the “Objectives” section of the abstract (92.4%). For the 7.6% of abstracts lacking this section, the full-text introduction was consulted. A foundational rule was established to assign only one primary SDG and one primary National Priority per thesis to prevent double-counting in the quantitative Thematic Alignment Index (TAI) calculation. Although a thesis could thematically relate to multiple goals, the panel determined the single most representative focus based on pre-defined principles, prioritizing health outcomes over social determinants when the latter were contextual factors.

Given the collaborative, real-time nature of the coding designed to achieve unanimity for all 6,157 cases, reliability was embedded in the consensus process itself and validated through two mechanisms:

1. **Pre-Coding Concordance Study:** A simple random sample of 250 theses confirmed a 92.4% agreement between the research focus stated in the abstract and the full-text body, validating the use of abstracts as a reliable source for coding.
2. **Classification Protocol Established:** Based on validation findings, the classification protocol was defined:

- Primary data source: Thesis title + Objectives section from abstract
- For incomplete abstracts: Full-text consultation to extract stated objectives from introduction or methods sections
- Assignment approach: Single assignment per thesis (one Sustainable Development Goal and one National Health Research Priority) to enable clear quantitative analysis without double-counting
- Classification Decision Framework: The classification was based on the thesis's primary stated objective.

All coding decisions were made unanimously. During sessions, any discrepancy in proposed codes among the four experts triggered immediate discussion. The panel re-examined the title and objective, applied the classification principles, and if necessary, consulted the full text until a consensus was reached. This ensured that every final assignment represented the collective expert judgment of the entire panel.

### 2.5 Keywords extraction

Peruvian thesis repositories require author-provided keywords as mandatory metadata. Peruvian theses include both Spanish abstracts and English abstracts per institutional requirements. Both languages were retained without translation to preserve semantic fidelity.

Prior to visualization analysis, keyword normalization was conducted using the Bibliometrix package in R (K-Synth Srl team, 2025). The preprocessing pipeline included conversion to lowercase, removal of English stopwords, elimination of generic and non-informative terms (e.g., thesis, study, research, university, Peru, year), and exclusion of keywords indicating the geographical location where the study was conducted, as these did not contribute to thematic interpretation. Punctuation marks (periods, commas, and semicolons) were removed, while hyphens in compound terms were preserved. Synonymous keywords were manually merged before analysis (e.g., “Diabetes type 2” and “Type II diabetes” into “type 2 diabetes”; “SARS-CoV-2” and “coronavirus” into “COVID-19”).

Keyword co-occurrence networks were constructed using VOSviewer (version 1.6.20) (Centre for Science Technology Studies, 2025). The full counting method was selected to preserve co-occurrence strength within the corpus (6,157 theses). A minimum keyword occurrence threshold of 25 was applied, corresponding to a 0.4% frequency cutoff, in order to balance thematic coverage and network interpretability (Van-Eck and Waltman, 2023). Association strength normalization was used for clustering, and the default resolution algorithm was applied. Edges were displayed only when a minimum of five co-occurrences was present.

In network visualizations, node size was proportional to keyword frequency, node color represented cluster membership, and edge thickness reflected co-occurrence strength. Overlay visualizations were generated to assess thematic maturity, with node color indicating the average publication year of each keyword (Van-Eck and Waltman, 2023). Density visualization confirmed a highly consolidated conceptual core centered on nursing care, health promotion, and quality of life, which interconnected the four thematic clusters identified in the co-occurrence network. Maternal–child health and chronic non-communicable diseases showed strong local density, whereas mental health and infection control occupied lower-density, peripheral positions, revealing structural gaps and opportunities for transdisciplinary research.

### 2.6 Thematic Alignment Index

The Thematic Alignment Index (TAI) (Chan and Reich, 2007) was used to quantify thematic coverage of undergraduate nursing theses in relation to the SDGs and Peru's National Health Research Priorities. The TAI represents the proportion of theses addressing a given SDG or national priority within the total analytic corpus. Importantly, the TAI does not measure research quality, methodological rigor, scientific impact, policy uptake, or implementation outcomes. Accordingly, it should be interpreted strictly as an indicator of thematic distribution rather than research performance or effectiveness.

Thematic alignment was determined through a correspondence and conceptual alignment analysis conducted by an expert panel. Classification was based primarily on the thesis title and the stated research objectives in the abstract. For theses lacking an explicit objectives section, the full-text introduction was consulted to identify the primary research focus. Each thesis was assigned to one primary SDG and one primary National Health Research Priority to prevent double-counting and to ensure comparability across categories.

The TAI was calculated with the formula:


$$\begin{array}{l} {\text{TAI}}_{\text{P}}=\frac{\text{Np}}{\text{Nt }}x\mathrm{100} \end{array}$$ (1)

Where *Np* represents the number of theses addressing a specific SDG or National Health Research Priority, and *Nt* denotes the total number of theses analyzed (*n* = 6 157). The index was computed independently for the 17 SDGs and the 53 National Health Research Priorities, enabling a quantitative assessment of thematic coverage across both international and national health agendas (MINSA, 2024).

TAI values were generated using RStudio (Posit Team, 2025) and are presented descriptively to support identification of thematic emphasis and potential research gaps. No formal concentration or inequality metrics were applied; therefore, observed distributions should be interpreted as descriptive patterns of thematic focus rather than inferential evidence of concentration.

## 3 Results

### 3.1 Temporal distribution of academic-scientific production

**Figure 2:**
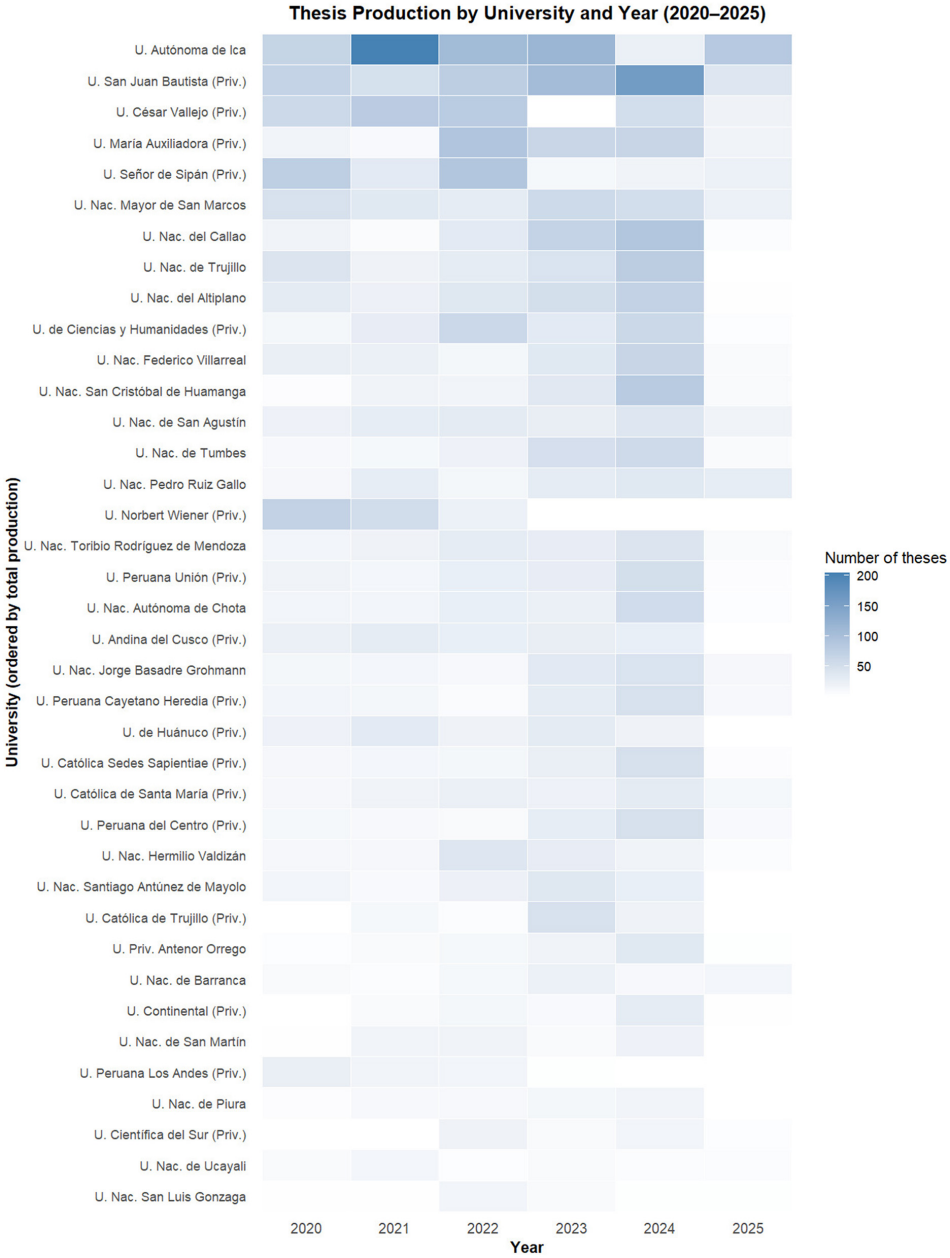
Thesis production by universities according to the years 2020–2025. U., University; Nac., National; Priv., Private.

Figure 2 displays the distribution of 6,157 undergraduate nursing theses produced by Peruvian universities between 2020 and 2025, disaggregated by institution and year. From a temporal perspective, thesis production appears more widely distributed across institutions during 2023 and 2024, with a greater number of universities exhibiting moderate-to-high intensity values in these years compared to 2020 and 2025, where production is visibly lower and more fragmented.

The heatmap reveals marked variation in thesis production across both dimensions. At the institutional level, Universidad Autónoma de Ica visually exhibits the highest concentration of thesis production, with a pronounced peak in 2021, representing the single most intense cell in the entire matrix. Elevated production levels for this institution are also visible in 2022 and 2023, followed by a lower but sustained output in subsequent years. Universidad San Juan Bautista exhibits a distinct peak in 2024, corresponding to one of the highest production intensities observed that year. Universidad César Vallejo and Universidad María Auxiliadora display moderate but sustained production across multiple years, particularly between 2021 and 2024.

Several public universities—including Universidad Nacional Mayor de San Marcos, Universidad Nacional del Callao, Universidad Nacional de Trujillo, and Universidad Nacional del Altiplano—demonstrate consistent but lower-intensity thesis production distributed across the study period, with visibly higher concentrations in 2023 and 2024. In contrast, a large number of institutions contribute small numbers of theses, often limited to 1 or 2 years, as indicated by low-intensity cells across the matrix.

### 3.2 Approach and type of applied research in theses

**Figure 3:**
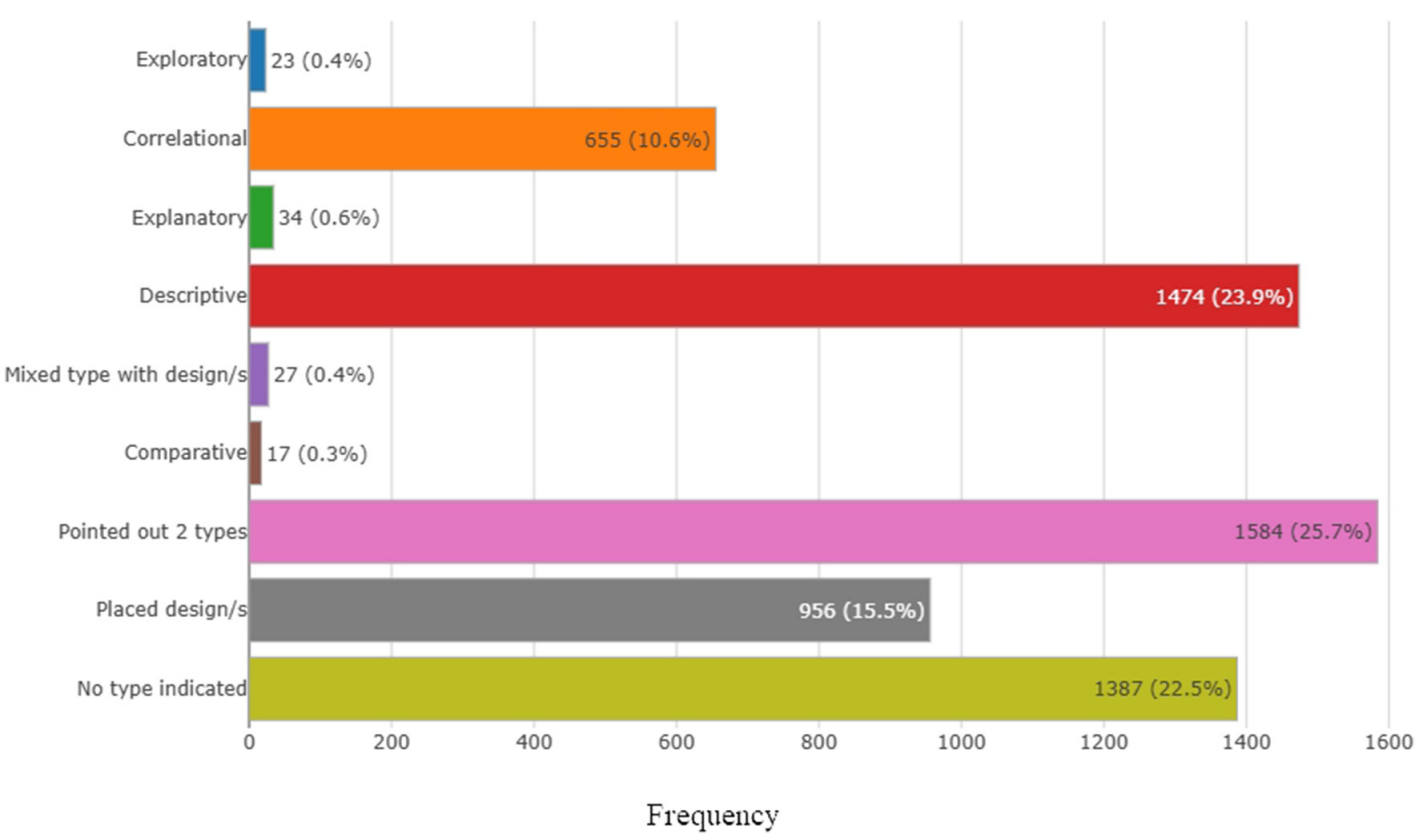
Theses by type of research.

Figure 3 presents the distribution of undergraduate nursing theses according to the type of research design reported. Among the 6 157 theses analyzed, the most frequently reported category was “Pointed out 2 types”, accounting for 1 584 theses (25.7%). This was followed by descriptive studies, which represented 1,474 theses (23.9%). A substantial proportion of theses did not explicitly specify the type of research design. Specifically, 1 387 theses (22.5%) were classified as “No type indicated”. Additionally, 956 theses (15.5%) were reported as having a placed or specified design without further classification.

Among studies that reported a single, clearly defined design, correlational studies accounted for 655 theses (10.6%). Less frequently reported designs included explanatory studies (34; 0.6%), exploratory studies (23; 0.4%), mixed-type studies with design specification (27; 0.4%), and comparative studies (17; 0.3%).

### 3.3 Thematic analysis and its contribution to the Sustainable Development Goals

**Figure 4:**
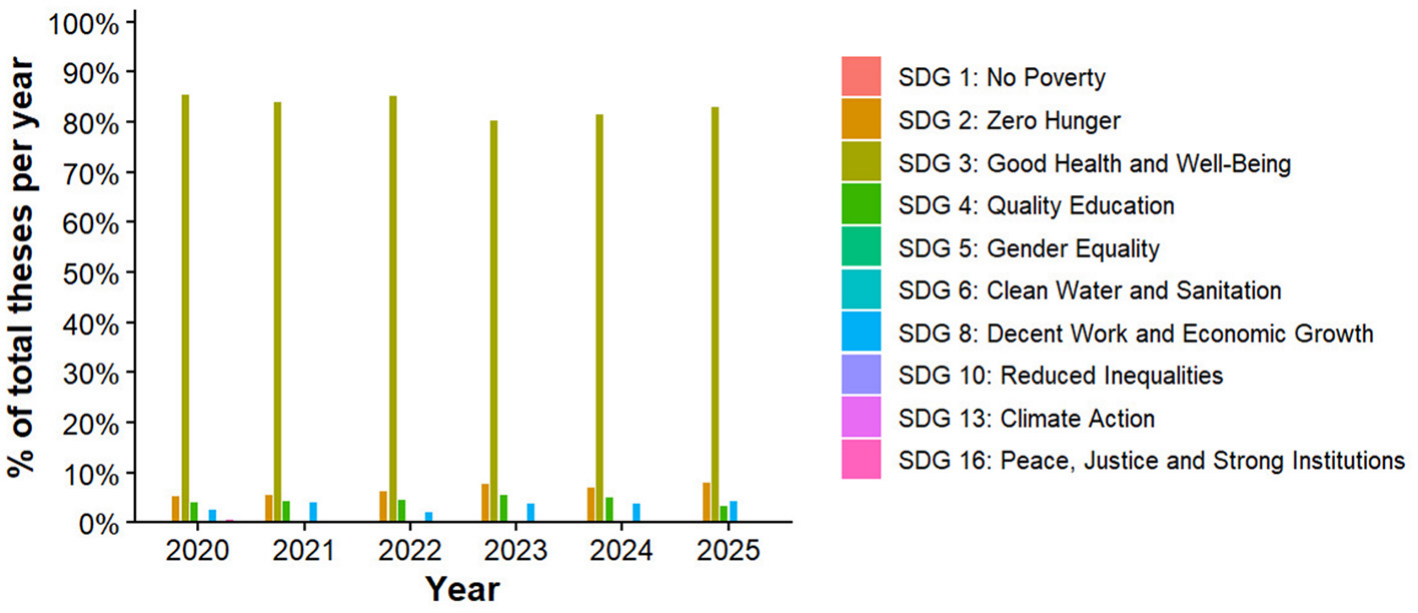
Relative contribution (%) of undergraduate nursing theses to the top 10 Sustainable Development Goals by year (2020–2025).

Figure 4 illustrates the relative contribution (%) of undergraduate nursing theses to the ten most frequently addressed Sustainable Development Goals (SDGs) between 2020 and 2025. Percentages were calculated within each year as the proportion of theses addressing a given SDG relative to the total annual thesis output (Table 1).

**Table 1:** Thematic alignment index by SDGs.

**Sustainable development goal**	**2020 (*n =* 880)**	**2021 (*n =* 1,112)**	**2022 (*n =* 1,268)**	**2023 (*n =* 1,420)**	**2024 (*n =* 1,114)**	**2025 (*n =* 371)**	**Total 2020–2025**
3: Good health and well-being	755 (85.8%)	945 (85.0%)	1,074 (84.7%)	1,157 (81.5%)	894 (80.3%)	289 (78.0%)	5,114 (83.06%)
2: Zero hunger	44 (5.0%)	69 (6.2%)	79 (6.2%)	103 (7.3%)	83 (7.5%)	44 (11.9%)	422 (6.85%)
4: Quality education	37 (4.2%)	50 (4.5%)	56 (4.4%)	76 (5.4%)	68 (6.1%)	18 (4.9%)	305 (4.95%)
8: Decent work and economic growth	30 (3.4%)	32 (2.9%)	42 (3.3%)	58 (4.1%)	48 (4.3%)	14 (3.8%)	224 (3.64%)
10: Reduced inequalities	3 (0.3%)	4 (0.4%)	2 (0.2%)	7 (0.5%)	6 (0.5%)	1 (0.3%)	23 (0.37%)
16: Peace, justice, and strong institutions	1 (0.1%)	4 (0.4%)	4 (0.3%)	5 (0.4%)	3 (0.3%)	1 (0.3%)	18 (0.29%)
5: Gender equality	3 (0.3%)	2 (0.2%)	3 (0.2%)	3 (0.2%)	3 (0.3%)	1 (0.3%)	15 (0.24%)
6: Clean water and sanitation	4 (0.5%)	3 (0.3%)	2 (0.2%)	2 (0.1%)	2 (0.2%)	1 (0.3%)	14 (0.23%)
1: No poverty	1 (0.1%)	1 (0.1%)	1 (0.1%)	2 (0.1%)	2 (0.2%)	0 (0.0%)	7 (0.11%)
13: Climate action	1 (0.1%)	1 (0.1%)	2 (0.2%)	2 (0.1%)	1 (0.1%)	0 (0.0%)	7 (0.11%)
12: Responsible consumption and production	0 (0.0%)	1 (0.1%)	1 (0.1%)	1 (0.1%)	0 (0.0%)	0 (0.0%)	3 (0.05%)
9: Industry, innovation, and infrastructure	0 (0.0%)	0 (0.0%)	1 (0.1%)	1 (0.1%)	0 (0.0%)	0 (0.0%)	2 (0.03%)
11: Sustainable cities and communities	0 (0.0%)	0 (0.0%)	1 (0.1%)	1 (0.1%)	0 (0.0%)	0 (0.0%)	2 (0.03%)
15: Life on land	1 (0.1%)	0 (0.0%)	0 (0.0%)	0 (0.0%)	0 (0.0%)	0 (0.0%)	1 (0.02%)
7: Affordable and clean energy	0 (0.0%)	0 (0.0%)	0 (0.0%)	0 (0.0%)	0 (0.0%)	0 (0.0%)	0 (0.00%)
14: Life below water	0 (0.0%)	0 (0.0%)	0 (0.0%)	0 (0.0%)	0 (0.0%)	0 (0.0%)	0 (0.00%)
17: Partnerships for the goals	0 (0.0%)	0 (0.0%)	0 (0.0%)	0 (0.0%)	0 (0.0%)	0 (0.0%)	0 (0.00%)

Values correspond to the Thematic Alignment Index (TAI), expressed as the percentage of theses per year addressing each SDG.

Across all years, SDG 3 (Good Health and Well-Being) overwhelmingly dominated, accounting for approximately 80–86% of theses annually. Although the absolute number of theses increased between 2020 and 2024, the relative contribution of SDG 3 remained consistently high, peaking in 2022 and 2024 and declining slightly in 2023 and 2025. Secondary SDGs—particularly SDG 2 (Zero Hunger) and SDG 4 (Quality Education)—represented 5–8% and 4–6% of annual theses, respectively. Their relative contributions showed a modest increase between 2020 and 2024, followed by a decrease in 2025, mirroring overall thesis production trends.

Other SDGs within the top ten, including SDG 8 (Decent Work and Economic Growth), SDG 10 (Reduced Inequalities), SDG 13 (Climate Action), and SDG 16 (Peace, Justice and Strong Institutions), consistently accounted for less than 5% of theses per year, indicating limited thematic diversification beyond core health-related priorities.

### 3.4 Thematic analysis and its contribution to health research priorities

**Figure 5:**
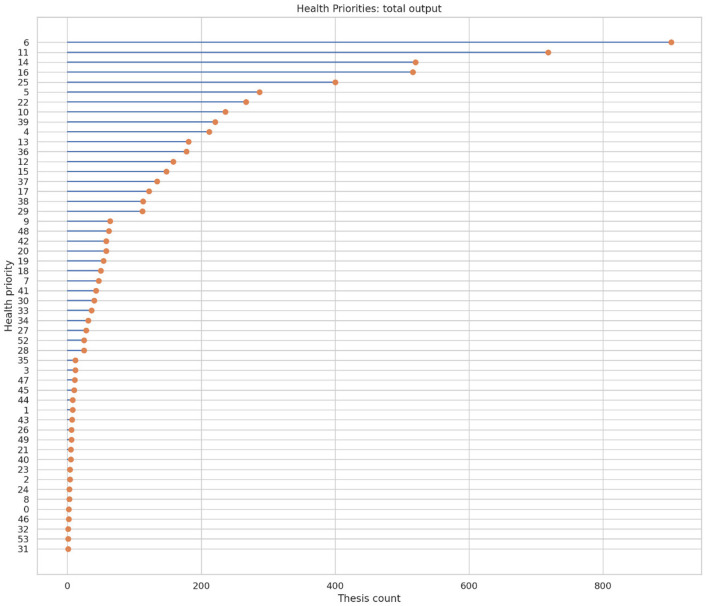
Thematic analysis and its contribution to Peruvian health research priorities. Priority numbers correspond to national health research priorities defined in Peru (MINSA, 2024).

Figure 5 presents the total number of undergraduate nursing theses classified according to Peru's National Health Research Priorities, as established in the Document N.° 184-2024-MINSA, Annex 1: “National Health Research Priorities 2024–2030”(MINSA, 2024). The values displayed correspond to absolute counts of theses assigned to each priority across the entire study period (2020–2025).

The numeric labels shown on the y-axis correspond to the official priority codes established in Annex 1 of the Ministerial Resolution (MINSA, 2024) and do not indicate rank or magnitude. Priorities in the figure are ordered in descending order according to cumulative thesis count, from top to bottom. Table 2 complements this visualization by reporting the exact number and percentage of theses corresponding to each priority, expressed as the Thematic Alignment Index (TAI). As shown in Figure 5 and detailed in Table 2, Priority 6 (Inadequate habits, customs, and lifestyles for good health) recorded the highest number of theses (*n* = 902; TAI = 14.65%), followed by Priority 11 (Malnutrition and anemia in the mother–child dyad; *n* = 718; TAI = 11.66%). Priority 14 (Mental and nervous system diseases) and Priority 16 (Cerebrovascular, cardiovascular, and metabolic diseases) accounted for 520 theses (TAI = 8.45%) and 516 theses (TAI = 8.38%), respectively. Priority 25 (COVID-19) represented 400 theses (TAI = 6.50%).

**Table 2:** Thematic alignment index by health priorities.

**Peruvian national health priority**	**2020 (*n =* 880)**	**2021 (*n =* 1,112)**	**2022 (*n =* 1,268)**	**2023 (*n =* 1,420)**	**2024 (*n =* 1,114)**	**2025 (*n =* 371)**	**Total 2020–2025**
6. Inadequate habits, customs, and lifestyles for good health	103 (11.7%)	171 (15.4%)	191 (15.1%)	218 (15.4%)	159 (14.3%)	60 (16.2%)	902 (14.65%)
11. Malnutrition and anemia (mother–child dyad)	76 (8.6%)	121 (10.9%)	144 (11.4%)	182 (12.8%)	148 (13.3%)	47 (12.7%)	718 (11.66%)
14. Mental and nervous system diseases	94 (10.7%)	96 (8.6%)	101 (8.0%)	117 (8.2%)	86 (7.7%)	26 (7.0%)	520 (8.45%)
16. Cerebrovascular, cardiovascular, and metabolic diseases	88 (10.0%)	93 (8.4%)	105 (8.3%)	114 (8.0%)	87 (7.8%)	29 (7.8%)	516 (8.38%)
25. COVID-19	121 (13.7%)	118 (10.6%)	68 (5.4%)	61 (4.3%)	25 (2.2%)	7 (1.9%)	400 (6.50%)
5. Low public awareness of mental health problems	38 (4.3%)	50 (4.5%)	59 (4.7%)	67 (4.7%)	54 (4.9%)	19 (5.1%)	287 (4.66%)
22. Non-communicable diseases in the elderly	32 (3.6%)	45 (4.0%)	55 (4.3%)	69 (4.9%)	50 (4.5%)	16 (4.3%)	267 (4.34%)
10. Exposure to unsafe conditions in work activities	30 (3.4%)	32 (2.9%)	42 (3.3%)	58 (4.1%)	48 (4.3%)	26 (7.0%)	236 (3.83%)
39. Low vaccination and immunization coverage	28 (3.2%)	42 (3.8%)	45 (3.5%)	51 (3.6%)	42 (3.8%)	13 (3.5%)	221 (3.59%)
4. Inadequate sexual and reproductive health	25 (2.8%)	36 (3.2%)	41 (3.2%)	52 (3.7%)	43 (3.9%)	15 (4.0%)	212 (3.44%)
13. Maternal, fetal, and neonatal complications	22 (2.5%)	30 (2.7%)	35 (2.8%)	44 (3.1%)	38 (3.4%)	12 (3.2%)	181 (2.94%)
36.Limited availability of human resources in health	24 (2.7%)	28 (2.5%)	34 (2.7%)	42 (3.0%)	38 (3.4%)	12 (3.2%)	178 (2.89%)
12.Malignant neoplasms (cancers)	19 (2.2%)	26 (2.3%)	31 (2.4%)	38 (2.7%)	33 (3.0%)	11 (3.0%)	158 (2.57%)
15. Tuberculosis	17 (1.9%)	24 (2.2%)	29 (2.3%)	36 (2.5%)	31 (2.8%)	11 (3.0%)	148 (2.40%)
37. Inequality in access to comprehensive care	16 (1.8%)	21 (1.9%)	26 (2.0%)	33 (2.3%)	28 (2.5%)	10 (2.7%)	134 (2.18%)
17. Sexually transmitted diseases and HIV/AIDS	14 (1.6%)	19 (1.7%)	24 (1.9%)	31 (2.2%)	26 (2.3%)	8 (2.2%)	122 (1.98%)
38. Limited availability of HR with competencies	13 (1.5%)	17 (1.5%)	22 (1.7%)	28 (2.0%)	24 (2.2%)	9 (2.4%)	113 (1.84%)
29. Healthcare-associated infections	13 (1.5%)	17 (1.5%)	22 (1.7%)	28 (2.0%)	23 (2.1%)	9 (2.4%)	112 (1.82%)
9. Low empowerment in the health system	7 (0.8%)	10 (0.9%)	12 (0.9%)	16 (1.1%)	14 (1.3%)	5 (1.3%)	64 (1.04%)
48. Scarce regulatory framework for bioethics	7 (0.8%)	9 (0.8%)	12 (0.9%)	15 (1.1%)	14 (1.3%)	5 (1.3%)	62 (1.01%)

Values correspond to the Thematic Alignment Index (TAI), expressed as the percentage of theses per year addressing each national health research priority. Priority numbers follow the official Peruvian classification (MINSA, 2024).

### 3.5 Keyword analysis and their alignment with Peru's public health challenges

**Figure 6:**
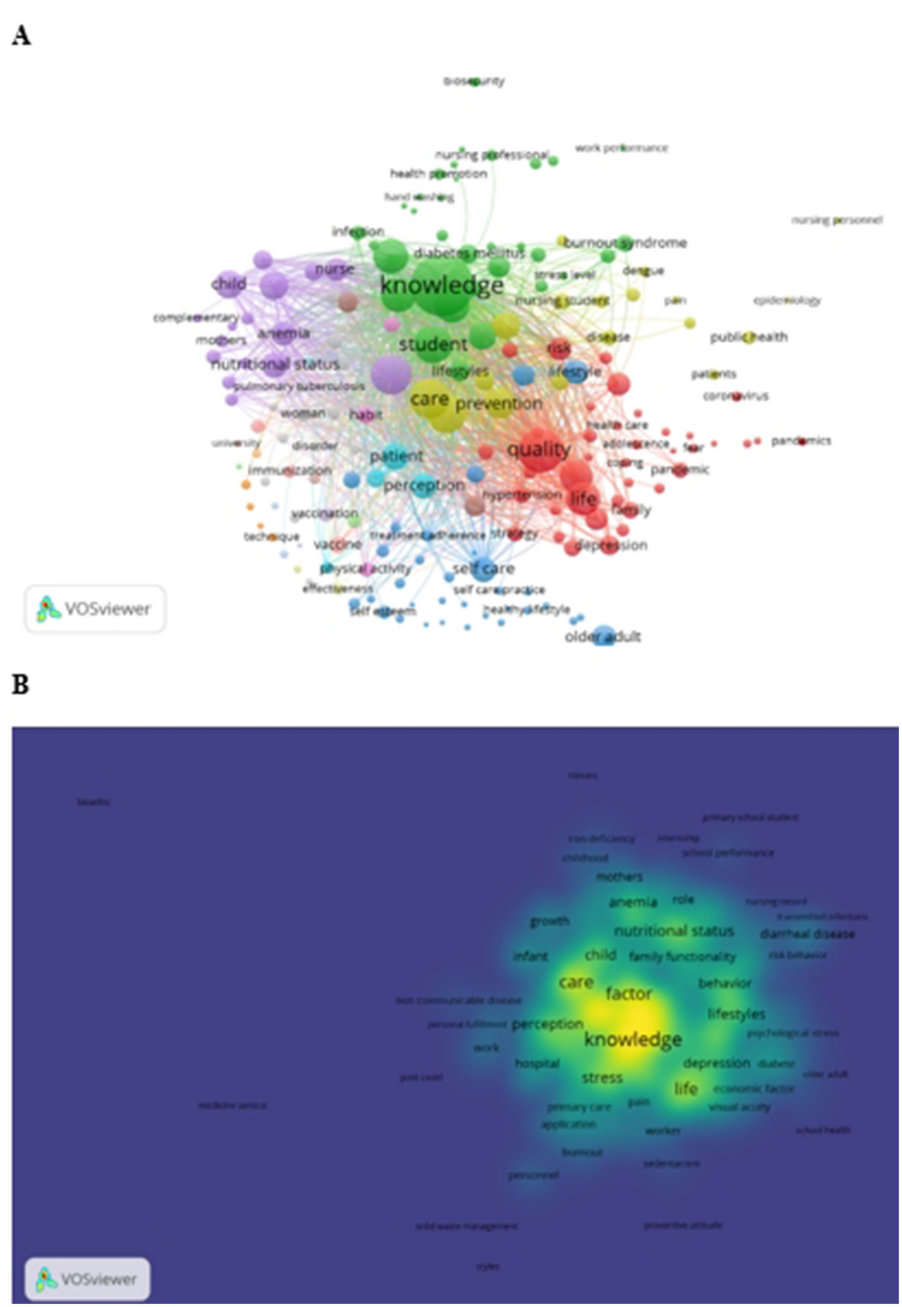
Visual analysis of keywords. **(A)** Keyword co-occurrence network of undergraduate nursing theses. **(B)** Keyword density map of undergraduate nursing theses.

The bibliometric analysis of keyword co-occurrence reveals a polynuclear structure in Peruvian nursing research, organized into four distinct thematic clusters (Figure 6A). The chronic non-communicable disease cluster (represented in blue) encompasses terms including “diabetes mellitus,” “hypertension,” and “lifestyle,” with spatial proximity indicating strong conceptual associations. A maternal-child health cluster (green) integrates concepts such as “anemia,” “breastfeeding,” and “malnutrition,” demonstrating thematic consolidation. The mental health cluster (yellow), containing terms like “anxiety,” “depression,” and “stress,” occupies a relatively peripheral position, suggesting limited integration with other domains. Meanwhile, the infection control cluster (red) addresses emerging priorities through terms including “COVID-19,” “biosafety,” and “vaccination.”

Notably, the largest nodes correspond to transversal concepts such as “nursing care,” “health promotion,” and “quality of life,” which function as conceptual bridges connecting specialized clusters. The density map (Figure 6B) confirms thematic consolidation around these core concepts. In this representation, color intensity indicates the local density of frequently co-occurring keywords, with warmer colors denoting higher densities. The highest-density region is centered on keywords such as nursing, care, knowledge, and quality of life. Lower-density regions appear toward the periphery of the network, where keywords are smaller and exhibit fewer co-occurrence links.

**Figure 7:**
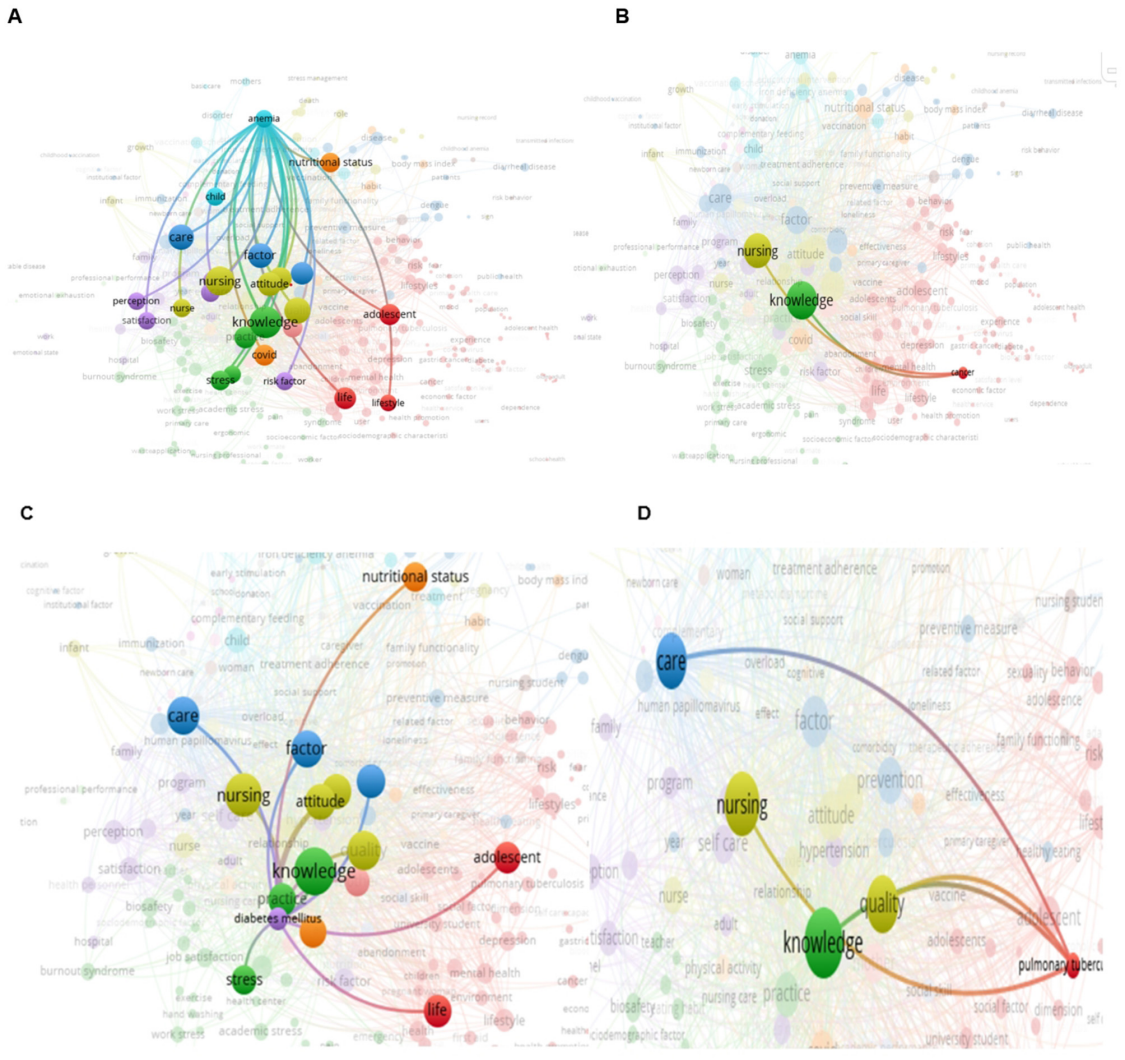
Visual analysis of keywords and their alignment with Peru's public health challenges. **(A)** Keyword co-occurrence network for anemia-related research. **(B)** Keyword co-occurrence network for cancer-related research. **(C)** Keyword co-occurrence network for diabetes mellitus-related research. **(D)** Keyword co-occurrence network for pulmonary tuberculosis-related research.

The visual analysis presented in Figures 7A–D demonstrates a focused investigation into how Peruvian nursing theses address specific national health challenges through their keyword selection and conceptual associations. The intentional selection of four key health problems—anemia (A), cancer (B), diabetes mellitus (C), and pulmonary tuberculosis (D)—reveals distinct patterns in research approaches and thematic focus. Figure 7A (Anemia) illustrates that research on this condition predominantly clusters around concepts such as “child nutrition,” “breastfeeding,” “pregnancy,” and “prevention”. Figure 7B (Cancer) shows connections to terminology including “palliative care,” “quality of life,” “pain management,” and “family support.” This pattern indicates a research focus oriented toward symptom management and supportive care rather than prevention or early detection.

Figure 7C (Diabetes Mellitus) reveals strong linkages to “self-care,” “education,” “physical activity,” and “adherence to treatment.” Figure 7D (Pulmonary Tuberculosis) demonstrates associations with “treatment adherence,” “directly observed therapy,” “prevention,” and “public health.”

### 3.6 Analysis of thematic coverage and identification of research gaps

The distribution of undergraduate nursing theses across the 17 Sustainable Development Goals (SDGs) is presented in Table 1. SDG 3 (Good Health and Well-being) accounted for 83.06% of all theses analyzed. SDG 2 (Zero Hunger) and SDG 4 (Quality Education) represented 6.85% and 4.95% of theses, respectively. Together, these three SDGs accounted for 94.87% of the total corpus. The remaining SDGs each accounted for less than 4% of theses. Specifically, SDG 5 (Gender Equality) represented 0.24%, SDG 6 (Clean Water and Sanitation) 0.23%, and SDG 13 (Climate Action) 0.11% of the total. No theses were classified under SDG 7 (Affordable and Clean Energy), SDG 14 (Life Below Water), or SDG 17 (Partnerships for the Goals).

The distribution of theses across Peru's National Health Research Priorities is shown in Table 2. The five most frequently addressed priorities accounted for 49.63% of all theses. Priority 6 (Inadequate habits, customs, and lifestyles for good health) represented 14.65%, followed by Priority 11 (Maternal–child malnutrition and anemia) at 11.66%. Priorities 14 (Mental and nervous system diseases) and 16 (Cerebrovascular, cardiovascular, and metabolic diseases) accounted for 8.45% and 8.38%, respectively. Priority 25 (COVID-19) represented 6.5% of theses. Several priorities accounted for less than 2% of theses, including Priority 9 (Social participation in the health system, 1.04%) and Priority 48 (Bioethics and ethical clinical research, 1.01%).

## 4 Discussion

The contemporary global landscape demands that university research transcend traditional academic boundaries to address pressing societal challenges with tangible, measurable impact (Belcher et al., 2020; Fecher and Hebing, 2021). This imperative becomes particularly acute in the health sector, where research must not merely generate knowledge but actively contribute to reducing morbidity, mortality, and health inequities (Costa et al., 2025). Within this context, the present study analyzed 6,157 undergraduate nursing theses produced in Peru between 2020 and 2025, revealing patterns of thematic concentration and methodological limitation that warrant careful interpretation.

The findings indicate that undergraduate nursing research in Peru is highly concentrated around a limited set of immediate health concerns, with 83.06% of theses aligned with SDG 3 (Good Health and Well-being) and nearly half addressing only five National Health Research Priorities. This concentration is not inherently unexpected, given that nursing is a health-centered discipline and that undergraduate research agendas are strongly shaped by curricular structures, clinical training environments, and feasibility constraints. The absence or minimal representation of theses aligned with SDGs such as Affordable and Clean Energy (SDG 7), Life Below Water (SDG 14), or Partnerships for the Goals (SDG 17) should not be interpreted uncritically as a shortcoming, as these goals are not traditionally nursing-dominant domains and often require interdisciplinary, infrastructural, or policy-oriented research approaches that exceed the scope and resources of undergraduate nursing education.

Nevertheless, the near absence of research addressing environmental determinants of health and social equity dimensions points to limited integration of broader social and environmental health frameworks within undergraduate nursing research training, a pattern consistent with prior evidence from Latin America indicating that undergraduate health research tends to prioritize proximal clinical and behavioral factors over structural determinants of health (Achury-Saldaña et al., 2022; Ñique et al., 2021; Gregorio-Chaviano et al., 2015).

When examined through Peru's official framework grouping National Health Research Priorities into three categories: diseases and health conditions, health services and systems, and living and working conditions (MINSA, 2024), the distribution reveals that undergraduate nursing research is predominantly concentrated in the first category. The most frequently addressed topics include health habits and lifestyles (Priority 6; 14.65%), maternal–child malnutrition and anemia (Priority 11; 11.66%), mental health disorders (Priority 14; 8.45%), and cardiovascular and metabolic diseases (Priority 16; 8.38%), areas that closely reflect nurses' clinical roles and prevailing training contexts.

In contrast, priorities related to health services organization, human resources, social participation, and ethical frameworks received substantially less attention, with social participation in health systems (Priority 9) and bioethics and clinical research (Priority 48) each accounting for approximately 1% of theses. This imbalance suggests that undergraduate nursing research remains largely focused on individual-level and clinical phenomena, with comparatively limited engagement in health systems research and broader structural determinants of health. Although this pattern is shaped by feasibility constraints and curricular orientation, it also identifies areas where undergraduate research training could be strengthened through incorporation of multidisciplinary or interprofessional research approaches (Farand et al., 2024; Dalton et al., 2021; Bourne et al., 2025).

The methodological profile reveals a clear predominance of descriptive and correlational designs, with 23.9% explicitly descriptive studies and 10.6% correlational studies. Notably, 22.5% of theses did not specify any research design, and an additional 25.7% reported more than one type without clear methodological differentiation. While this lack of specification may partly reflect variability in abstract quality and reporting practices—as the present analysis relied primarily on thesis abstracts supplemented by full-text consultation only when abstracts lacked objectives sections—the magnitude of this finding points to a broader structural weakness in undergraduate research training related to methodological clarity, conceptual rigor, and adherence to reporting standards.

Similar patterns have been reported in other Latin American contexts, where undergraduate health research tends to privilege feasibility and completion over methodological precision and analytical depth (Achury-Saldaña et al., 2022; Ñique et al., 2021). Descriptive research undoubtedly plays an essential role, particularly in contexts where routine health information systems are limited, establishing baseline data and characterizing health conditions (Wang et al., 2022; Hothersall, 2024). However, the scarcity of experimental, interventional, and implementation-oriented studies substantially limits the capacity of undergraduate research to inform practice change or health system improvement. As emphasized by Riegel et al. (2021), nursing science must move beyond describing phenomena toward testing interventions, evaluating outcomes, and supporting evidence implementation in real-world settings.

The keyword co-occurrence analysis revealed a polynuclear thematic structure organized into four main clusters—chronic non-communicable diseases, maternal–child health, mental health, and infection control—with transversal nodes such as “nursing,” “care,” “knowledge,” and “quality of life” indicating shared conceptual foundations. Nevertheless, the spatial separation between clusters suggests limited conceptual integration, particularly between mental health and chronic disease research, and between infection control and broader care domains. This fragmentation indicates that undergraduate nursing research is structured around discrete problem areas rather than integrated care perspectives. The disease-specific subnetworks for anemia, cancer, diabetes mellitus, and tuberculosis illustrate focused yet narrow conceptual architectures, each aligning with core nursing roles—prevention in anemia, supportive care in cancer, self-management in diabetes, and treatment adherence in tuberculosis—while simultaneously revealing limited incorporation of cross-cutting themes such as health systems integration, environmental determinants, or social vulnerability. The peripheral position of the mental health cluster highlights its weak integration with physical health research, despite strong evidence of bidirectional relationships between mental and chronic conditions (Sattar et al., 2023).

These findings align with international evidence that undergraduate health professions education requires fundamental reorientation to address social determinants of health, community needs, and health equity (Ashwell et al., 2025; Nguyen et al., 2025). International experiences suggest that this limitation is not inevitable. Atuyambe et al. (2016) documented how community-based education enabled medical students in Uganda to contribute directly to primary healthcare through water, sanitation, and hygiene education, while Nguyen et al. (2025) showed that early exposure to underserved communities during medical education improved students' preparedness to address health disparities. Hothersall (2024) describes curriculum reforms integrating public health, social determinants, and planetary health throughout medical education rather than as isolated topics. Strengthening undergraduate research relevance in Peru may require curricular strategies that explicitly incorporate social determinants of health, health systems research, and methodological training focused on clarity and feasibility of applied designs.

This study has limitations that should be acknowledged. The analysis was restricted to undergraduate nursing theses from SUNEDU-licensed universities in Peru; therefore, the findings do not reflect research production in non-licensed institutions or at other academic levels, such as specialization, master's, or doctoral programs. The bibliometric analyses were based on thesis metadata, titles, and abstracts, which may not fully capture the complete content or methodological quality of the research, although the reliance on abstracts was supported by prior concordance validation.

Thematic alignment with the Sustainable Development Goals and National Health Research Priorities was determined through expert panel consensus rather than standardized classification systems. Finally, the study focused on research production volume and thematic distribution, without assessing research quality, knowledge utilization, or policy impact.

Despite these limitations, the study demonstrates the feasibility of examining formative research alignment with national health agendas and provides a quantitative framework that may be transferable to other disciplines and contexts characterized by limited research investment and mandatory undergraduate thesis requirements.

Future research should explore factors influencing thesis topic selection, track graduates' research utilization and career trajectories longitudinally, conduct comparative analyses across health professions, test curricular reforms through intervention studies, and investigate mechanisms to strengthen the translation of undergraduate research into practice and policy.

## 5 Conclusions

This bibliometric analysis describes the thematic distribution, methodological characteristics, and conceptual structure of 6,157 undergraduate nursing theses produced in Peru between 2020 and 2025. The findings show a strong concentration of research in health-related topics, particularly those aligned with SDG 3 (Good Health and Well-being), and a more balanced distribution across Peru's National Health Research Priorities, reflecting nursing's disciplinary focus on clinical and public health problems. The analysis also indicates a predominance of descriptive and correlational study designs, together with considerable variability in methodological reporting. Keyword co-occurrence mapping identified four main thematic clusters—chronic non-communicable diseases, maternal–child health, mental health, and infection control—suggesting that undergraduate nursing research is organized around distinct problem areas with limited conceptual integration.

Overall, this study provides a descriptive overview of undergraduate nursing research trends in Peru, offering a structured reference for understanding how formative research topics and methods have evolved in recent years.

## Data Availability

The datasets presented in this study can be found in online repositories. The names of the repository/repositories and accession number(s) can be found below: Mendeley data doi: 10.17632/sz6nx423h6.1.
