# Supplementary material for: Are we researching the right questions? Bibliometric analysis of undergraduate nursing thesis alignment with Peru's health priorities
Source: Front Res Metr Anal. 2026 Feb 12;11:1738032. doi: 10.3389/frma.2026.1738032 (PMC12935872; doi:10.3389/frma.2026.1738032)
Supplement: Supplementary file 1 [file Table_1.docx]

**National Health Research Priority**

1. Deficient access to safe water and basic sanitation
2. Effects of climate change with an emphasis on the El Niño phenomenon
3. Environmental pollution and population exposure to heavy metals, metalloids, and other hazardous products
4. Inadequate sexual and reproductive health with an emphasis on adolescent pregnancy
5. Low public awareness of mental health problems
6. Inadequate habits, customs, and lifestyles for good health
7. Social exclusion and poor quality of life for people with disabilities
8. Underutilization of renewable energy
9. Low empowerment, inclusion, and participation of social actors in the health system with an emphasis on Integrated Health Networks
10. Exposure to unsafe conditions in work activities
11. Malnutrition and anemia due to deficits in the mother-child dyad during pregnancy and up to 36 months of the child's age
12. Malignant neoplasms (Cancers)
13. Maternal, fetal, and neonatal complications, morbidity, and mortality
14. Mental and nervous system diseases with an emphasis on: brain degeneration, unipolar depression, post-traumatic stress from disasters and violence, alcoholism and drug addiction, and in the workplace
15. Tuberculosis
16. Cerebrovascular, cardiovascular, and metabolic diseases, with an emphasis on arterial hypertension, diabetes mellitus, obesity, and other types of overnutrition
17. Sexually transmitted diseases and HIV/AIDS
18. Acute diarrheal diseases, intestinal and parasitic diseases
19. Vector-borne and zoonotic diseases, with an emphasis on dengue, zika, and leishmaniasis
20. Acute respiratory infections and pneumonias
21. Land transport accidents
22. Non-communicable diseases in the elderly
23. Dental diseases
24. Interstitial lung diseases
25. COVID-19
26. Viral hepatitis B, cirrhosis, and other chronic liver diseases
27. Trauma from external causes
28. Urinary tract diseases
29. Healthcare-associated infections
30. Musculoskeletal diseases in workers
31. Mycosis (Fungal infections)
32. Drowning accidents or accidents that obstruct breathing
33. Deficient infrastructure and equipment of public health services for comprehensive care with an emphasis on people with diabetes, cancer, rare and orphan diseases, and disabilities, as well as for emergency and disaster response
34. Deficient supply of the service portfolio, medicines, and supplies for comprehensive care with an emphasis on people with diabetes, cancer, rare and orphan diseases, and disabilities, as well as for emergency and disaster response
35. Limited approach to man-made contaminants in water, soil, air, and food
36. Limited availability (gap), high turnover of human resources in health, poor working conditions for human resources in health
37. Inequality in access to comprehensive care in health services for vulnerable populations, with an emphasis on the elderly, people with disabilities, people affected by heavy metal and other chemical contamination, as well as incarcerated individuals and their minor companions
38. Limited availability of human resources with competencies for comprehensive care with an emphasis on mental health, disability, as well as emergencies and disasters
39. Low vaccination and immunization coverage
40. Weak intersectoral and intergovernmental coordination among actors of the national health system
41. Limited response capacity in emergency and disaster situations and poor implementation of health risk management
42. Scarce mental health interventions with a comprehensive, intercultural, universal, and community-based approach for the prevention, diagnosis, and timely care of cases
43. Deficient monitoring of necessary pharmaceuticals, medical devices, and biomedical equipment for emergency care
44. Deficient referral and counter-referral process
45. Scarce implementation of the digital government policy with an emphasis on data exchange between the Minsa-EsSalud healthcare information system platforms and the Integrated Health Networks
46. Fragmented, inadequate, and insufficient financing of public health services, with an emphasis on mental health and the incarcerated population and their minor companions
47. Deficient regulation and oversight of medicines by the corresponding authorities
48. Scarce regulatory framework that promotes Bioethics and high-quality ethical clinical research in the country, with an emphasis on the production of vaccines and rapid diagnostic methods in a pandemic
49. Delays in granting sanitary registration for products with health impact
50. Inefficiency in the disability certification process, with an emphasis on the standards used for its classification
51. Limited reach of the International Classification of Functioning, Disability and Health (ICF) at the national level
52. Scarce regulatory framework for citizen care and well-being with an emphasis on active breaks and regulated physical activity
53. Regulatory gap for the formulation of repellents

Retrieved from: MINSA. Resolución Ministerial 184-2024/MINSA Prioridades Nacionales en salud 2024-2030 [Internet]. 2024. Recuperado a partir de: https://www.gob.pe/institucion/minsa/normas-legales/5364816-184-2024-minsa

Traduction: ChatGpt Ia V 5.2, and validate by researches.
