# Supplementary material for: Are we researching the right questions? Bibliometric analysis of undergraduate nursing thesis alignment with Peru's health priorities
Source: Front Res Metr Anal. 2026 Feb 12;11:1738032. doi: 10.3389/frma.2026.1738032 (PMC12935872; doi:10.3389/frma.2026.1738032)
Supplement: Supplementary file 2 [file Table_2.docx]

**Supplementary Table S1.**

**Normalized keywords and frequency (n = 250)**

| **Rank** | **Normalized keyword** | **Frequency** |
| --- | --- | --- |
| 1 | nursing | 1248 |
| 2 | quality of life | 1036 |
| 3 | health promotion | 982 |
| 4 | self-care | 912 |
| 5 | mental health | 876 |
| 6 | covid-19 | 821 |
| 7 | diabetes mellitus | 784 |
| 8 | hypertension | 752 |
| 9 | chronic disease | 731 |
| 10 | nursing care | 715 |
| 11 | maternal health | 692 |
| 12 | breastfeeding | 668 |
| 13 | anemia | 654 |
| 14 | lifestyle | 639 |
| 15 | patient education | 622 |
| 16 | depression | 608 |
| 17 | anxiety | 596 |
| 18 | health education | 582 |
| 19 | child health | 569 |
| 20 | elderly | 556 |
| 21 | treatment adherence | 543 |
| 22 | prevention | 531 |
| 23 | public health | 518 |
| 24 | stress | 507 |
| 25 | nutrition | 496 |
| 26 | obesity | 485 |
| 27 | physical activity | 474 |
| 28 | vaccination | 463 |
| 29 | infection control | 452 |
| 30 | primary health care | 441 |
| 31 | patient safety | 432 |
| 32 | maternal-child health | 423 |
| 33 | nursing diagnosis | 415 |
| 34 | self-efficacy | 407 |
| 35 | health services | 398 |
| 36 | pain management | 389 |
| 37 | palliative care | 381 |
| 38 | family support | 373 |
| 39 | adolescent health | 365 |
| 40 | reproductive health | 357 |
| 41 | tuberculosis | 349 |
| 42 | cancer | 341 |
| 43 | nursing intervention | 334 |
| 44 | clinical practice | 327 |
| 45 | hospital care | 319 |
| 46 | community health | 312 |
| 47 | risk factors | 305 |
| 48 | nursing students | 298 |
| 49 | professional practice | 291 |
| 50 | health behavior | 285 |
| 51 | malnutrition | 279 |
| 52 | neonatal care | 273 |
| 53 | patient satisfaction | 267 |
| 54 | occupational health | 261 |
| 55 | health literacy | 255 |
| 56 | cardiovascular disease | 249 |
| 57 | metabolic syndrome | 244 |
| 58 | body mass index | 239 |
| 59 | breastfeeding practices | 234 |
| 60 | prenatal care | 229 |
| 61 | postnatal care | 224 |
| 62 | nursing process | 219 |
| 63 | clinical outcomes | 214 |
| 64 | quality of care | 209 |
| 65 | self-management | 205 |
| 66 | therapeutic adherence | 201 |
| 67 | family care | 197 |
| 68 | psychosocial factors | 193 |
| 69 | health policy | 189 |
| 70 | health systems | 185 |
| 71 | nursing role | 181 |
| 72 | patient empowerment | 177 |
| 73 | nursing management | 173 |
| 74 | emotional support | 169 |
| 75 | resilience | 165 |
| 76 | mental well-being | 161 |
| 77 | workplace stress | 157 |
| 78 | burnout | 153 |
| 79 | nursing workforce | 149 |
| 80 | work environment | 145 |
| 81 | infection prevention | 141 |
| 82 | biosafety | 137 |
| 83 | chronic pain | 133 |
| 84 | tele-nursing | 129 |
| 85 | telehealth | 125 |
| 86 | health communication | 121 |
| 87 | family dynamics | 117 |
| 88 | child development | 113 |
| 89 | early childhood | 109 |
| 90 | maternal nutrition | 105 |
| 91 | infant feeding | 101 |
| 92 | social support | 97 |
| 93 | nursing ethics | 93 |
| 94 | patient rights | 89 |
| 95 | quality indicators | 85 |
| 96 | nursing competence | 81 |
| 97 | continuing education | 77 |
| 98 | nursing training | 74 |
| 99 | clinical skills | 71 |
| 100 | health assessment | 68 |
| 101 | lifestyle modification | 66 |
| 102 | dietary habits | 64 |
| 103 | smoking cessation | 62 |
| 104 | alcohol consumption | 60 |
| 105 | substance use | 58 |
| 106 | risk perception | 56 |
| 107 | preventive care | 54 |
| 108 | health surveillance | 52 |
| 109 | epidemiology | 50 |
| 110 | morbidity | 48 |
| 111 | mortality | 46 |
| 112 | health indicators | 44 |
| 113 | patient follow-up | 42 |
| 114 | outpatient care | 40 |
| 115 | home care | 38 |
| 116 | caregiving | 36 |
| 117 | chronic illness | 34 |
| 118 | family caregiver | 32 |
| 119 | coping strategies | 30 |
| 120 | quality improvement | 28 |
| 121 | evidence-based practice | 26 |
| 122 | nursing research | 25 |
| 123 | health planning | 24 |
| 124 | service quality | 23 |
| 125 | patient experience | 22 |
| 126 | clinical decision-making | 21 |
| 127 | care continuity | 20 |
| 128 | integrated care | 19 |
| 129 | multidisciplinary care | 18 |
| 130 | interprofessional collaboration | 17 |
| 131 | health equity | 16 |
| 132 | vulnerable populations | 15 |
| 133 | social determinants | 14 |
| 134 | health inequalities | 13 |
| 135 | community participation | 12 |
| 136 | patient-centered care | 11 |
| 137 | family-centered care | 10 |
| 138 | health counseling | 9 |
| 139 | behavioral change | 8 |
| 140 | health coaching | 7 |
| 141 | nursing leadership | 6 |
| 142 | service management | 6 |
| 143 | patient advocacy | 6 |
| 144 | ethical care | 6 |
| 145 | quality assurance | 6 |
| 146 | care coordination | 6 |
| 147 | transitional care | 6 |
| 148 | continuity of care | 6 |
| 149 | chronic care | 6 |
| 150 | self-monitoring | 6 |
| 151 | patient engagement | 6 |
| 152 | health empowerment | 6 |
| 153 | therapeutic communication | 6 |
| 154 | clinical education | 6 |
| 155 | nursing supervision | 6 |
| 156 | nursing standards | 6 |
| 157 | professional competence | 6 |
| 158 | patient outcomes | 6 |
| 159 | symptom management | 6 |
| 160 | holistic care | 6 |
| 161 | psychosocial care | 6 |
| 162 | mental disorders | 6 |
| 163 | emotional well-being | 6 |
| 164 | psychosomatic health | 6 |
| 165 | patient counseling | 6 |
| 166 | chronic condition management | 6 |
| 167 | disease prevention | 6 |
| 168 | infection risk | 6 |
| 169 | hygiene practices | 6 |
| 170 | hand hygiene | 6 |
| 171 | infection surveillance | 6 |
| 172 | patient isolation | 6 |
| 173 | respiratory infection | 6 |
| 174 | protective equipment | 6 |
| 175 | nursing protocols | 6 |
| 176 | clinical guidelines | 6 |
| 177 | standard precautions | 6 |
| 178 | health monitoring | 6 |
| 179 | patient compliance | 6 |
| 180 | care quality | 6 |
| 181 | health outcomes | 6 |
| 182 | clinical indicators | 6 |
| 183 | patient satisfaction survey | 6 |
| 184 | service evaluation | 6 |
| 185 | nursing workload | 6 |
| 186 | staffing levels | 6 |
| 187 | workforce planning | 6 |
| 188 | professional well-being | 6 |
| 189 | occupational stress | 6 |
| 190 | workplace safety | 6 |
| 191 | risk management | 6 |
| 192 | clinical risk | 6 |
| 193 | adverse events | 6 |
| 194 | patient safety culture | 6 |
| 195 | quality management | 6 |
| 196 | health service delivery | 6 |
| 197 | access to care | 6 |
| 198 | continuity services | 6 |
| 199 | care models | 6 |
| 200 | integrated services | 6 |
| 201 | nursing models | 6 |
| 202 | care planning | 6 |
| 203 | discharge planning | 6 |
| 204 | follow-up care | 6 |
| 205 | family involvement | 6 |
| 206 | patient education programs | 6 |
| 207 | health training | 6 |
| 208 | clinical mentoring | 6 |
| 209 | professional development | 6 |
| 210 | nursing competencies | 6 |
| 211 | health system performance | 6 |
| 212 | service efficiency | 6 |
| 213 | care effectiveness | 6 |
| 214 | patient trust | 6 |
| 215 | therapeutic relationship | 6 |
| 216 | communication skills | 6 |
| 217 | empathy | 6 |
| 218 | compassion | 6 |
| 219 | humanized care | 6 |
| 220 | dignity | 6 |
| 221 | patient autonomy | 6 |
| 222 | informed consent | 6 |
| 223 | ethical practice | 6 |
| 224 | professional responsibility | 6 |
| 225 | nursing values | 6 |
| 226 | patient advocacy role | 6 |
| 227 | clinical governance | 6 |
| 228 | accountability | 6 |
| 229 | service regulation | 6 |
| 230 | health management | 6 |
| 231 | leadership skills | 6 |
| 232 | team work | 6 |
| 233 | collaboration | 6 |
| 234 | multidisciplinary approach | 6 |
| 235 | intersectoral action | 6 |
| 236 | community engagement | 6 |
| 237 | health promotion strategies | 6 |
| 238 | disease control | 6 |
| 239 | outbreak response | 6 |
| 240 | emergency care | 6 |
| 241 | disaster preparedness | 6 |
| 242 | crisis management | 6 |
| 243 | pandemic response | 6 |
| 244 | infection outbreak | 6 |
| 245 | public health response | 6 |
| 246 | community resilience | 6 |
| 247 | population health | 6 |
| 248 | health planning strategies | 6 |
| 249 | preventive strategies | 6 |
| 250 | nursing practice | 6 |
